# Lesion-specific cortical activation following sensory stimulation in patients with subacute stroke

**DOI:** 10.1186/s12984-023-01276-8

**Published:** 2023-11-13

**Authors:** Wei Li, Chong Li, Aixian Liu, Ping-Ju Lin, Linhong Mo, Hongliang Zhao, Quan Xu, Xiangzun Meng, Linhong Ji

**Affiliations:** 1 Division of Intelligent and Biomechanical System, Department of Mechanical Engineering, Tsinghua University, Haidian, Beijing, China; 2 School of Clinical Medicine, Tsinghua Medicine, Tsinghua University, Beijing, China; 3 Medical Research Center, Beijing Tsinghua Changgung Hospital, Tsinghua University, Beijing, China; 4 Neurological Rehabilitation Center, Beijing Rehabilitation Hospital Affiliated to Capital Medical University, Beijing, China; 5 Department of Rehabilitation Medicine, Beijing Tsinghua Changgung Hospital, Tsinghua University, Beijing, China; 6 Department of Radiology, Beijing Tsinghua Changgung Hospital, Tsinghua University, Beijing, China; 7 State Key Laboratory of Multimodal Artificial Intelligence Systems, Institute of Automation, Chinese Academy of Sciences, Beijing, China

**Keywords:** Sensory stimulation, Stroke, EEG, Brain lesion profiles, Primary sensorimotor cortex

## Abstract

**Background:**

Sensory stimulation can play a fundamental role in the activation of the primary sensorimotor cortex (S1-M1), which can promote motor learning and M1 plasticity in stroke patients. However, studies have focused mainly on investigating the influence of brain lesion profiles on the activation patterns of S1-M1 during motor tasks instead of sensory tasks. Therefore, the objective of this study is to explore the lesion-specific activation patterns due to different brain lesion profiles and types during focal vibration (FV).

**Methods:**

In total 52 subacute stroke patients were recruited in this clinical experiment, including patients with basal ganglia hemorrhage/ischemia, brainstem ischemia, other subcortical ischemia, cortical ischemia, and mixed cortical–subcortical ischemia. Electroencephalograms (EEG) were recorded following a resting state lasting for 4 min and three sessions of FV. FV was applied over the muscle belly of the affected limb’s biceps for 3 min each session. Beta motor-related EEG power desynchronization overlying S1-M1 was used to indicate the activation of S1-M1, while the laterality coefficient (LC) of the activation of S1-M1 was used to assess the interhemispheric asymmetry of brain activation.

**Results:**

(1) Regarding brain lesion profiles, FV could lead to the significant activation of bilateral S1-M1 in patients with basal ganglia ischemia and other subcortical ischemia. The activation of ipsilesional S1-M1 in patients with brainstem ischemia was higher than that in patients with cortical ischemia. No activation of S1-M1 was observed in patients with lesions involving cortical regions. (2) Regarding brain lesion types, FV could induce the activation of bilateral S1-M1 in patients with basal ganglia hemorrhage, which was significantly higher than that in patients with basal ganglia ischemia. Additionally, LC showed no significant correlation with the modified Barthel index (MBI) in all patients, but a positive correlation with MBI in patients with basal ganglia lesions.

**Conclusions:**

These results reveal that sensory stimulation can induce lesion-specific activation patterns of S1-M1. This indicates FV could be applied in a personalized manner based on the lesion-specific activation of S1-M1 in stroke patients with different lesion profiles and types. Our study may contribute to a better understanding of the underlying mechanisms of cortical reorganization.

**Supplementary Information:**

The online version contains supplementary material available at 10.1186/s12984-023-01276-8.

## Introduction

Up to 85% of stroke survivors encounter motor deficits [1]. Despite the recovery, more than 50% of patients are left with a residual motor deficit [2]. This high percentage of stroke patients with a residual motor deficit following recovery can be ascribed to the following facts: (1) lack of new therapies that improve rehabilitation [3]; and (2) individual variability in stroke recovery [4]. The problem of individual variability can lead to difficulty to make the choice of treatment for clinicians. Despite this, clinical trials are often designed with a “one size fits all” point of view regardless of clinical profile, and finally, some patients cannot benefit from the designed clinical trial [5]. Besides, several studies have demonstrated that the recovery outcome can also be correlated with the clinical profile, such as lesion location and lesion type [6–8].

Regarding lesion locations, several studies have found that patients with subcortical stroke have greater improvement in motor recovery of upper extremities than patients with cortical stroke after repetitive transcranial magnetic stimulation [9, 10] and patients with cortical–subcortical stroke after three rehabilitation training sessions [11]. Moreover, patients with lesions involving different cortical–subcortical areas showed different motor recovery outcomes. Specifically, injuries to the internal/external capsule, corpus callosum, and optical radiation are associated with good outcomes, in contrast to lesions of the corona radiata supraventricular [12]. Regarding lesion types, several studies have revealed that patients with hemorrhagic stroke showed greater functional improvement in the early phase of rehabilitation than those with ischemic stroke [13, 14]. However, the different results have also been reported. For example, in one study no significant difference in rehabilitation outcomes between patients with ischemic and hemorrhagic stroke was observed [15]. Similar functional improvements in gait and posture outcomes between ischemic and hemorrhagic stroke after conventional physiotherapy combined with robotic-assisted gait therapy have been observed [7]. The differences in motor recovery outcomes can be linked to the lesion-specific reorganization patterns of the sensorimotor cortex, including the reorganization of the contralesional sensorimotor cortex [16, 17], the reorganization of the ipsilesional sensorimotor cortex [18], and the reorganization of the bilateral sensorimotor cortex [19].

The activation of the sensorimotor cortex may play an important role in the functional reorganization of the sensorimotor cortex. Some researchers have demonstrated that the activation patterns (e.g., activation regions and activation magnitude) of the sensorimotor cortex in stroke patients can depend on the lesion profiles using functional magnetic resonance imaging (fMRI). For example, the activation of the contralesional sensorimotor cortex for cortical stroke patients, as well as the activation of the bilateral sensorimotor cortex for subcortical stroke patients, occurred during paretic limb movement [20], whereas activation of the ipsilesional somatosensory association cortex was observed only in patients with subcortical stroke following touch discrimination training, but not in patients with lesions involving cortical regions [21]. Besides, the activation of the contralesional sensorimotor cortex in patients with subcortical stroke was higher than that in cortical stroke following repetitive hand movements based on fMRI [22]. One electroencephalography (EEG) study has also revealed that subcortical stroke patients had stronger activation of the sensorimotor cortex than cortex stroke patients during movement attempts and active movement [23]. Therefore, patients with cortical stroke and subcortical stroke showed different activation patterns of the sensorimotor cortex. However, most studies have focused on the effect of lesion locations in chronic stroke patients on the activation of the sensorimotor cortex following motor tasks using fMRI. Few studies have reported on the effects of lesion profiles in subacute stroke patients on the activation of the sensorimotor cortex using EEG following sensory stimulation.

Sensory stimulation can contribute greatly to promoting the plasticity of the motor cortex and enhancing motor learning [24, 25]. Focal vibration (FV) and electric stimulation are two of the commonly used sensory stimulations to improve the limb function of patients with neurological disorders. Different from electric stimulation, FV is a natural way of sensory stimulation [26] and can provide selective excitation of the proprioceptive input to the central nervous system by primarily targeting Ia-afferent. The ability to manipulate proprioceptive afferents can contribute to the recovery of altered proprioception in stroke patients, thus leading to the improvement of the ability of planning and control during limb movement [27]. Generally, FV with a frequency of 50 to 120 Hz and an amplitude of 0.2 to 4 mm can activate the spindle afferents preferentially by stretching muscle fibers and then traveling the proprioceptive information along the upper body proprioceptive pathway, up to the primary sensory cortex (S1), and the primary motor cortex (M1) [28]. Some studies have demonstrated that FV can have an effect on improving motor function in some patients with stroke, spinal cord injury, Parkinson, and multiple sclerosis by the reorganization of the sensorimotor cortex [29]. For example, FV can improve arm stability [30], improve gait performance [28], and alleviate upper limb spasticity [31, 32]. The rehabilitation effect on the mitigation of spasticity and the improvement in motor function was associated with the reshaping of corticospinal plasticity and the reorganization of the motor cortex [31, 32]. Besides, FV integrated with motor training can have a greater effect on the improvement of motor function and the reorganization of sensorimotor cortical compared with motor training alone [32]. Therefore, it is very important to explore how the clinical profile of stroke patients affects the activation of the sensorimotor cortex following FV, which can contribute to a better understanding of mechanisms underlying cortical reorganization in heterogeneous stroke patients. However, current studies have focused mainly on exploring the activation of the sensorimotor cortex induced by FV using fMRI and EEG in both healthy subjects and stroke patients [33]. The variations of the FV-induced activation of the sensorimotor cortex depending on brain lesion locations and types have not been fully understood.

Actually, the combination of brain lesion size and location, which was reflected in the concept of “brain lesion profile,” should be focused on, rather than size or location individually [34]. Therefore, the effect of brain lesion profile on the activation of S1-M1 induced by FV in subacute stroke patients is explored in our study. In our study, stroke patients with different brain lesion profiles included patients with basal ganglia ischemia, brainstem ischemia, other subcortical ischemia (only referring to subcortical lesion locations except for basal ganglia and brainstem), cortical ischemia, and mixed (referred to as cortical–subcortical) ischemia. Besides, the occurrence of hemorrhagic strokes (10.1%) was lower than that of ischemic strokes (89.9%) [35], while the most common sites of bleeding were the basal ganglia (50%). Obviously, hemorrhage in basal ganglia occurred more often than in other locations. Based on this, patients with hemorrhage/ischemia only in the basal ganglia was taken as an example to investigate the effects of brain lesion types on the activation of S1-M1 in subacute stroke patients induced by FV.  In summary, the objective of this study is to investigate the effects of brain lesion profiles and brain lesion types on the activation of S1-M1 in subacute stroke patients induced by FV, which can contribute to having a good knowledge of lesion-specific cortical activation following sensory stimulation. The following hypothesis is put forward: the activation patterns of S1-M1 induced by FV depended on brain lesion profiles and lesion types, in which the activation of S1-M1 in patients with other subcortical strokes (basal ganglia, brainstem, other subcortical regions) induced by FV might be higher than that in patients with cortical stroke (pure cortical regions, mixed cortical–subcortical regions) and the activation of S1-M1 in patients with basal ganglia hemorrhage induced by FV might be higher than that in patients with basal ganglia ischemia.

## Material and methods

### Subjects and experimental paradigm

**Fig. 1:**
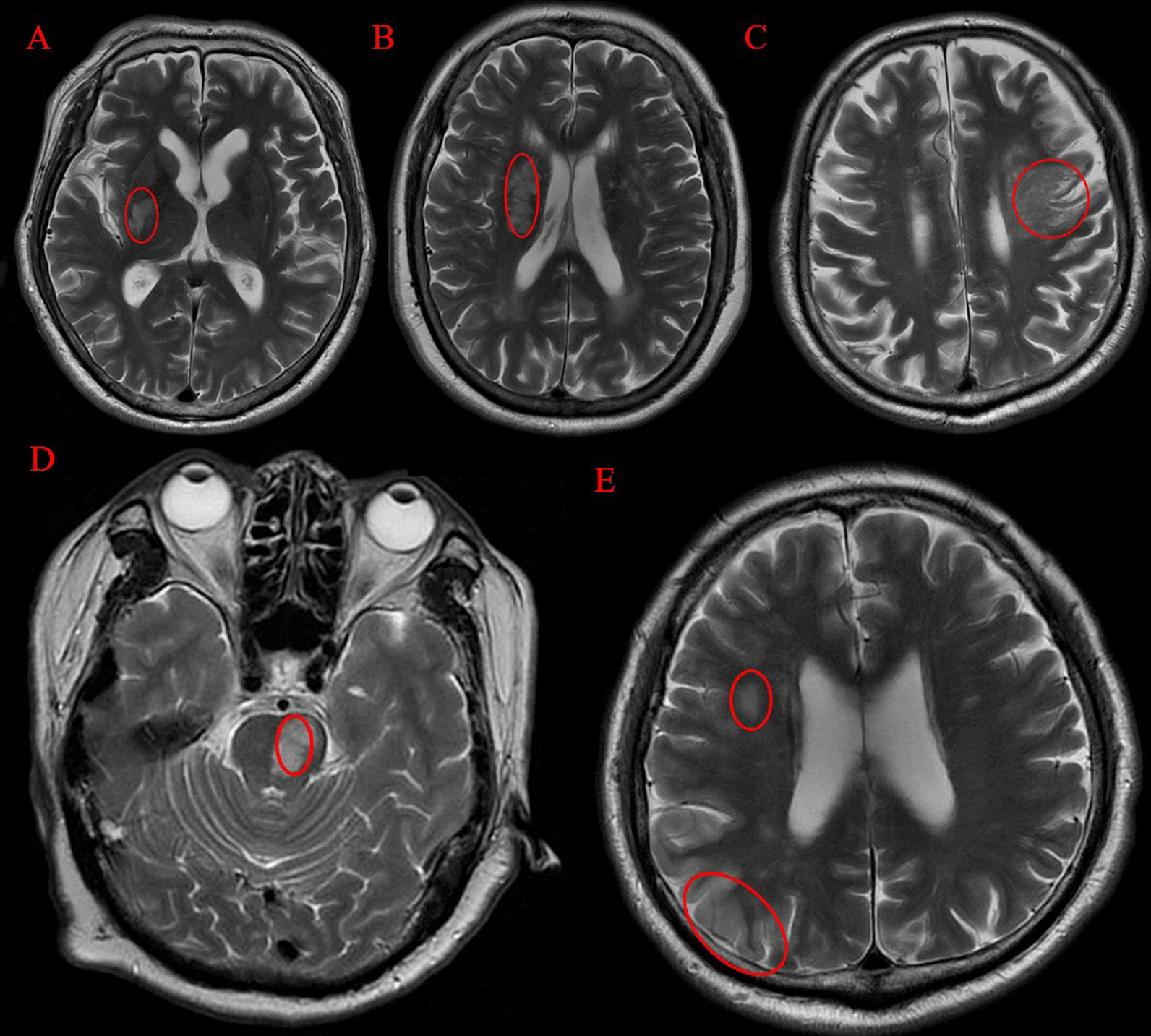
The lesion locations of brain lesion profiles, including basal ganglia (**A**), other subcortical regions (e.g. lateral ventricle) (**B**), cortical region (**C**), brainstem (**D**), and mixed cortical–subcortical regions (**E**), were marked with a red ellipse

In total 52 subacute stroke patients, including 9 patients with basal ganglia hemorrhage, 13 patients with basal ganglia ischemia, 8 patients with brainstem ischemia, 7 patients with other subcortical ischemia, 9 patients with cortical stroke, and 6 patients with mixed ischemia, were recruited from Shenzhen Nanshan District People's Hospital, Beijing Tsinghua Changgung Hospital, and Beijing Rehabilitation Hospital Affiliated with Capital Medical University, respectively. The classification of stroke lesion location was based on structural MRI (e.g., T1-weighted) by expert neuroradiologists from the respective hospitals (Fig. 1). The clinical characteristics of each type of stroke patient are shown in Table 1. The modified Barthel index (MBI), which is a commonly used scale to assess independence in activities of daily living of stroke survivors, was assessed by licensed occupational or physical therapists from the respective hospitals based on the standard training program provided by the KOSCO study. The MBI score ranges from 0 and 100, with higher MBI scores indicating greater independence. The inclusion criteria were as follows: (1) age ranging from 18 to 80 years; (2) less than 6 months after first-onset stroke; (3) no history of other neurological diseases, such as psychiatric disorders and traumatic brain injury; (4) the ability to understand the tester’s requests; (5) no abnormalities except for hemorrhagic or ischemic lesion based on conventional MRI. The exclusion criteria were as follows: (1) serious complications of the lung, heart, and kidney; (2) the severely restricted movement of joints due to pain, pronged joint immobilization, and a piece of torn cartilage. All patients provided written informed consent before participation. Experiments were conducted in accordance with the Declaration of Helsinki. This study was approved by the local ethics committee, including the Medical Ethics Committee of Shenzhen Nanshan District People’s Hospital (2018-0210-2), Beijing Tsinghua Changgung Hospital (19075-0-01), and Beijing Rehabilitation Hospital Affiliated to Capital Medical University (209bkkj-028).

**Table 1:** The clinical characteristics of each type of stroke patients

Group	Sex	Age (mean ± SD)	Lesion type	lesion location	Months Since Stroke	MBI (mean ± SD)
1	4 F/5 M	57.8 ± 9.3	Hemorrhage	Basal ganglia	1.6 ± 0.9	46.1 ± 24.9
2	2F/11 M	58.4 ± 7.4	Ischemia	Basal ganglia	2.8 ± 1.5	66.8 ± 21.8
3	1 F/7 M	61.4 ± 7.8	Ischemia	Brainstem	2.4 ± 1.9	49.9 ± 13.5
4	2 F/5 M	60.5 ± 21.0	Ischemia	Other subcortical regions	2 ± 1.4	53 ± 33.2
5	1 F/8 M	50.9 ± 16.4	Ischemia	Cortical regions	2.9 ± 1.5	41.7 ± 16.6
6	6 M	55.7 ± 8.2	Ischemia	Mixed regions	1.9 ± 1.6	53.2 ± 26.0

*F* female, *M* male, *SD* standard deviation, *MBI* modified Barthel index

**Fig. 2:**
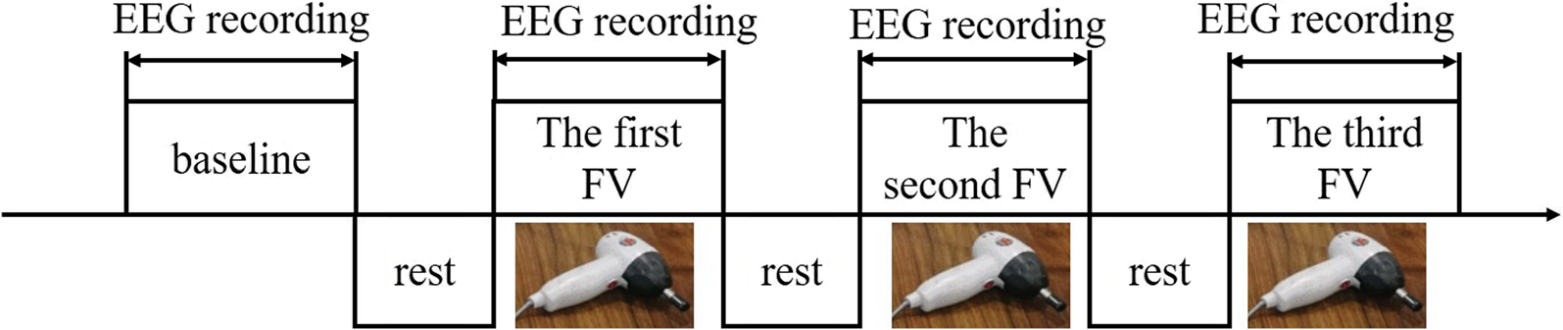
The diagram of the experimental process. The inset is the mechanical vibratory device used in our study

All the subjects were requested to sit with the upper limbs relaxed. FV (frequency: 75 Hz; amplitude: 1.2 mm), which was generated by a mechanical vibration device with a vibration head with a diameter of 17 mm (YS-889, Jialemei Health Care Co., Ltd., Taiwan, China, see Fig. 2), was applied over the biceps brachii’s muscle belly of the affected upper extremity of each patient. The experiment was carried out according to the following phases (Fig. 2): (1) baseline (S0), the resting-state EEG lasting for 4 min was recorded; (2) the first FV stimulation phase (S1), EEG was recorded lasting for 3 min following the first FV stimulation; (3) the second FV stimulation phase (S2), the same as S1; (4) the third FV stimulation phase (S3), the same as S1. An interval of 5 min between phases was required. While EEG signals were recorded, all subjects were requested to keep their eyes closed. To reduce the interference with EEG signals from physiological signals, patients were asked to minimize movement of the head, eyes, body, and refrain from chewing.

### EEG recordings and signal processing

EEG signals were recorded using the Waveguard system with standard 32 Ag/AgCl electrodes EEG cap (ANT Neuro, Netherlands). In this system, the CPz electrode was chosen as the reference electrode and the ground reference laid between Fz and FPz electrodes. During EEG recording, the electrode impedances were set below 5 kΩ.

The filtering of EEG data was carried out using EEGLAB, in which the 0.5 Hz low-pass filter was applied first, and then the 45 Hz high-pass filter was applied. A common average reference was chosen. EEG data were divided into segments of 2 s. The EEG data with an amplitude exceeding ± 70 μV were removed by visual inspection. Physiological signals from the body itself, such as eye blinking, muscle activity, and the heartbeat, were removed by an independent component analysis method.

It is well known that beta (13–30 Hz) frequency-band oscillations (~ 13–30 Hz) are observed over the sensorimotor cortex and are more prominent during sensorimotor processes. Several studies have also shown that low-beta, mid-beta (18–21 Hz), and high-beta might reflect a widespread cortical activation of sensorimotor systems, the activation of the motor cortex, and the activation of task-specific neural circuits with a more circumscribed cortical representation, respectively [36, 37]. Besides, based on two previous studies [38, 39], the sub-beta frequencies, in which FV can induce motor-related power desynchronization (MRPD) in subacute stroke patients, were chosen in this study: beta1 (13–18 Hz), beta2 (18–21 Hz), and beta3 (21–30 Hz). In the power analysis, we calculated first the power spectral density of each segment using the Welch method (pWelch algorithm, 0.5 Hz frequency resolution, an overlapping 1-s Hanning window, no phase shift) and summed up all the segments. Second, we averaged all the segments. Finally, the power was estimated as follows:

$${\text{RP}}(f1,f2) = \frac{{\int_{f1}^{f2} {PSD(f1,f2)df} }}{{\int_{0.5}^{45} {PSD(0.5,45)df} }}$$

where ${\text{RP(f1,f2)}}$ and ${\text{PSD(f1,f2)}}$ were the relative power and the power spectral density in the frequency band [f1, f2], respectively. To determine whether the differences in the activation of S1-M1 among different types of patients existed, beta MRPD overlying S1-M1, which reflected the activation of S1-M1, was used [38]. Beta MRPD was expressed by the negative results of the formula, reflecting a percentage decrease in the beta power during the task when compared with the resting-state (baseline) condition. A greater magnitude of MRPD overlying the sensorimotor cortex meant a higher activation of the sensorimotor cortex. MRPD induced by FV each time was estimated as follows:

$${\text{MRP}}{{\text{D}}_{\{ {\text{S1}},{\text{S2}},{\text{S3}}\} }} = \frac{{{\text{R}}{{\text{P}}_{_{\{ {\text{S1,S2,S3}}\} }}} - {\text{R}}{{\text{P}}_{_{{\text{Baseline}}}}}}}{{{\text{R}}{{\text{P}}_{{\text{Baseline}}}}}} \times 100\%$$

In our study, the average MRPD induced by FV three times was calculated. Based on a previous study [40], the average laterality coefficient (LC) was computed as follows:

$${\text{LC}} = \frac{{\sum\limits_{S1}^{S3} {(MRPD_{{\{ S1,S2,S3\} }}^{{\text{Ipsilesional S1 - M1}}} - MRPD_{{\{ S1,S2,S3\} }}^{{\text{Contralesional S1 - M1}}} )} }}{3}$$

### Statistical analysis

All statistical analyses were carried out in IBM SPSS Statistics 27. The Kruskal–Wallis H test (one-way non-parametric analysis of variance [ANOVA]) was carried out to determine whether age, time since stroke, and MBI were significantly different among stroke patients with different brain lesion profile and lesion types. In the EEG statistical analysis, beta1, beta2, and beta3 were considered independent variables. To test whether FV led to a significant activation of S1-M1 in stroke patients, a three-way ANOVA for repeated measures (hereafter referred to as three-way ANOVA) was carried out as the main statistical procedure, in which time factors (baseline and during FV) and location factors (contralesional S1-M1 and ipsilesional S1-M1) were considered within-subject factors, and group factors (basal ganglia hemorrhage/ischemia, brainstem ischemia, subcortical ischemia, cortical ischemia, and mixed ischemia) were treated as between-subject factors. When the interaction was statistically significant, post hoc comparisons were carried out.

To test the significance of differences in the activation of S1-M1 among stroke patients, one-way non-parametric ANOVA (two independent samples: Mann–Whitney U test; multiple independent samples: Kruskal–Wallis H test) was carried out as the main statistical procedure, in which location factors (contralesional S1-M1 and ipsilesional S1-M1) were considered within-subject factors, and group factors (lesion type group: basal ganglia hemorrhage/ischemia; lesion location group: basal ganglia ischemia, brainstem ischemia, other subcortical ischemia, cortical ischemia, and mixed ischemia) were treated as between-subject factors. If the main factors or their interactions showed significance, post hoc comparisons were carried out.

During ANOVA testing, the adjustment was made to the degrees of freedom if the assumption of sphericity checked by Mauchly’s test was of significance. Considering that the sample size was small and that basal ganglia stroke was more common compared with other regions’ strokes, Bayesian Pearson correlation analysis was used to determine whether these indices, including the activation of ipsilesional S1-M1, the activation of contralesional S1-M1, and LC, had a correlation with MBI scores, only for all the stroke patients and patients with basal ganglia lesions. Due to the robustness of the ANOVA under the application of non-normally distributed data [41], the Shapiro–Wilk test was used to examine whether these metrics were normally distributed only for Bayesian Pearson correlation analysis. In the post hoc comparisons, the false discovery rate was used in multiple hypothesis testing to correct for multiple comparisons. Significance was defined as *p* < 0.05.

## Results

The Kruskal–Wallis H test indicated that age, time since stroke, and MBI of stroke patients with different lesion locations were not significantly different (p = 0.530, 0.547, 0.611).

### The changes of power in stroke patients with different brain lesion profiles and brain lesion types

**Fig. 3:**
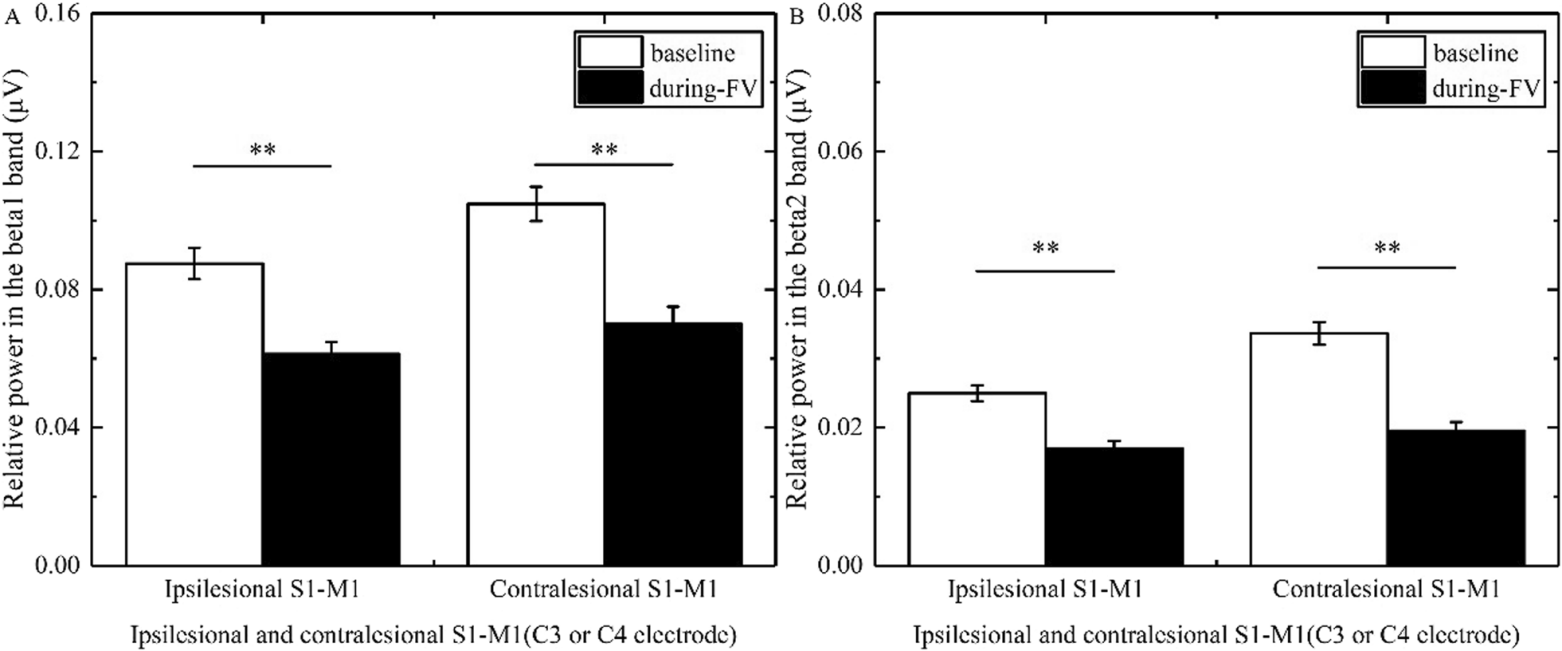
Comparison of relative power in the different bands overlying S1-M1 between baseline and during-FV (Focal vibration) in patients with basal ganglia hemorrhage. **A** shows the significant results of relative power overlying ipsilesional or contralesional S1-M1 during FV compared with the baseline phase in the beta1 band. **B** shows the significant results of relative power overlying ipsilesional or contralesional S1-M1 during FV compared with the baseline phase in the beta2 band. (Asterisks indicate significant differences; *0.01 ≤ *P* < 0.05, **P < 0.01)

**Fig. 4:**
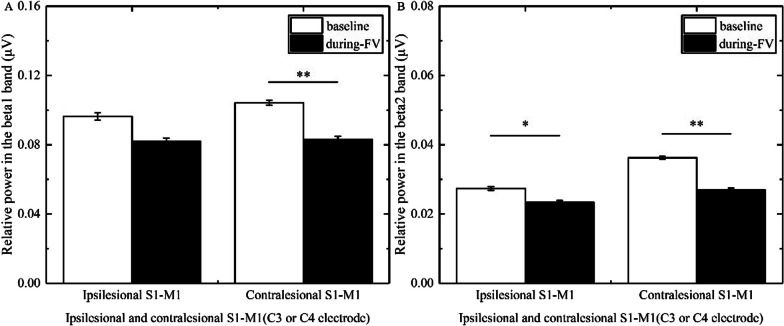
Comparison of relative power in the different bands overlying S1-M1 between baseline and during-FV (Focal vibration) in patients with basal ganglia ischemia. **A** shows the significant results of relative power overlying contralesional S1-M1 during FV compared with the baseline phase in the beta1 band. **B** shows the significant results of relative power overlying ipsilesional or contralesional S1-M1 during FV compared with the baseline phase in the beta2 band. (Asterisks indicate significant differences; *0.01 ≤ *P* < 0.05, **P < 0.01)

**Fig. 5:**
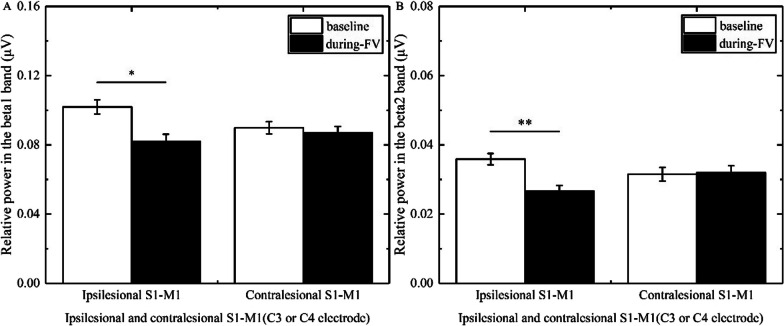
Comparison of relative power in the different bands overlying S1-M1 between baseline and during-FV (Focal vibration) in patients with brainstem ischemia. **A** shows the significant results of relative power overlying ipsilesional S1-M1 during FV compared with the baseline phase in the beta1 band. **B** shows the significant results of relative power overlying ipsilesional S1-M1 during FV compared with the baseline phase in the beta2 band. (Asterisks indicate significant differences; *0.01 ≤ *P* < 0.05, **P < 0.01)

In the beta1 band, three-way ANOVA illustrated that the main effect time [F(1, 46) = 23.964, p < 0.001, η^2^ = 0.343] and the group × time interaction [F(5, 46) = 2.925, p = 0.022, η^2^ = 0.241] had a significance; the main effect location and the group × location, location × time, and location × time × group interactions had no significance. The post hoc test revealed that FV led to a significant reduction of power overlying bilateral S1-M1 (ipsilesional S1-M1, p = 0.006, Cohen’d = 1.127; contralesional S1-M1, p < 0.0001, Cohen’d = 2.736) in patients with basal ganglia hemorrhage (Fig. 3A), a significant reduction of power overlying contralesional S1-M1 (p = 0.006, Cohen’d = 0.922) in patients with basal ganglia ischemia (Fig. 4A), and a significant reduction of power overlying ipsilesional S1-M1 (p = 0.014, Cohen’d = 1.075) in patients with brainstem ischemia (Fig. 5A).

**Fig. 6:**
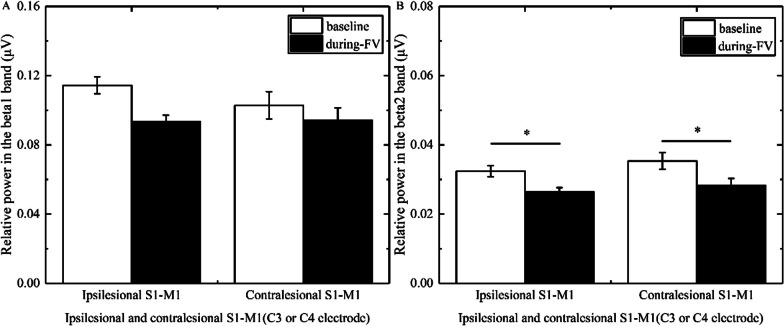
Comparison of relative power in the different bands overlying S1-M1 between baseline and during-FV (Focal vibration) in patients with other subcortical ischemia. **A** shows no significant results of relative power overlying ipsilesional or contralesional S1-M1 during FV compared with the baseline phase in the beta1 band. **B** shows the significant results of relative power overlying ipsilesional or contralesional S1-M1 during FV compared with the baseline phase in the beta2 band. (Asterisks indicate significant differences; *0.01 ≤ *P* < 0.05, **P < 0.01)

In the beta2 band, three-way ANOVA showed that the main effect location [F(1, 46) = 19.318, p < 0.001, η^2^ = 0.296], the main effect time [F(1, 46) = 22.734, p < 0.001, η^2^ = 0.331], and the group × time interaction [F(5, 46) = 2.546, p = 0.041, η^2^ = 0.217] had a significance; the location × time, location × group, and group × location × time interactions had no significance. The post hoc test revealed that FV led to a significant reduction in power overlying bilateral S1-M1 (ipsilesional S1-M1, p = 0.008, Cohen’d = 1.167; contralesional S1-M1, p = 0.002, Cohen’d = 1.670) in patients with basal ganglia hemorrhage (Fig. 3B), a significant reduction in power overlying bilateral S1-M1 (ipsilesional S1-M1, p = 0.027, Cohen’d = 0.637; contralesional S1-M1, p = 0.008, Cohen’d = 0.921) in patients with basal ganglia ischemia (Fig. 4B), a significant reduction of power overlying ipsilesional S1-M1 (p < 0.0001, Cohen’d = 2.530) in patients with brainstem ischemia (Fig. 5B), and a significant reduction in power overlying bilateral S1-M1 (ipsilesional S1-M1, p = 0.034, Cohen’d = 0.878; contralesional S1-M1, p = 0.027, Cohen’d = 1.003) in patients with other subcortical ischemia (Fig. 6B).

In the beta3 band, three-way ANOVA showed that the main effect location [F(1, 46) = 16.789, p = 0.002, η^2^ = 0.267], the main effect time [F(1, 46) = 17.654, p < 0.0001, η^2^ = 0.277], and the location × group interaction [F(4, 46) = 2.460, p = 0.047, η^2^ = 0.211] had a significance; the time × group [F(5, 46) = 2.105, p = 0.082, η^2^ = 0.185], location × time [F(1, 46) = 2.523, p = 0.119, η^2^ = 0.052], and location × time × group interactions [F(5, 46) = 2.229, p = 0.067, η^2^ = 0.195] had no significance. Post hoc tests revealed that the power of ipsilesional S1-M1 was significantly lower than that of contralesional S1-M1 in the baseline phase in patients with basal ganglia hemorrhage (p = 0.034, Cohen’d = 0.914, Additional file 1: Fig. S1A), patients with basal ganglia ischemia (p = 0.034 Cohen’d = 0.696, Additional file 1: Fig. S1B), patients with brainstem ischemia (p = 0.038, Cohen’d = 0.858, Additional file 1: Fig. S1C), and patients with cortical ischemia (p = 0.036, Cohen’d = 0.841, Additional file 1: Fig. S1D), while the power of ipsilesional S1-M1 was significantly lower than the power of contralesional S1-M1 in the FV phase in patients with cortical ischemia (p = 0.005, Cohen’d = 1.462, Additional file 1: Fig. S1D) and patients with mixed cortical–subcortical ischemia (p = 0.005, Cohen’d = 2.656, Additional file 1: Fig. S1E).

### The differences in MRPD among stroke patients with different brain lesion profiles and brain lesion types

**Fig. 7:**
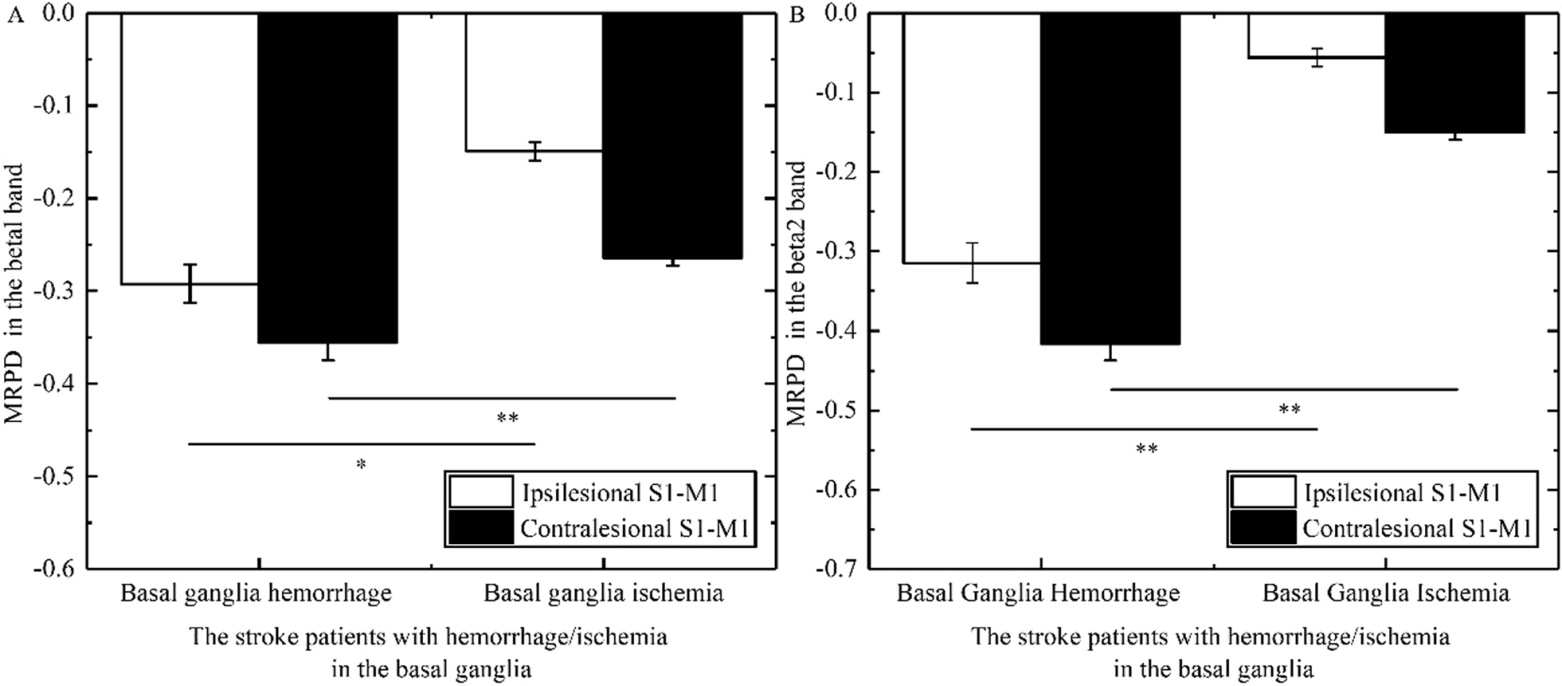
Comparison of MRPD (Motor-related power desynchronization) in the different bands induced by FV (Focal vibration) overlying S1-M1 in the stroke patients with between basal ganglia hemorrhage and basal ganglia ischemia. **A** shows the significant difference in MRPD of contralesional or ipsilesional S1-M1 between stroke patients with basal ganglia hemorrhage and that with basal ganglia ischemia in the beta1 band. **B** shows the significant difference in MRPD of contralesional or ipsilesional S1-M1 between stroke patients with basal ganglia hemorrhage and that with basal ganglia ischemia in the beta2 band. (Asterisks indicate significant differences; *0.01 ≤ *P* < 0.05, **P < 0.01)

In the beta1 band, the Mann–Whitney U test revealed that MRPD of bilateral S1-M1 in patients with basal ganglia hemorrhage was lower than that in patients with basal ganglia ischemia (ipsilesional S1-M1, p = 0.021; contralesional S1-M1, p = 0.0056, Fig. 7A). In the beta2 band, the Mann–Whitney U test revealed that MRPD of bilateral S1-M1 in patients with basal ganglia hemorrhage was lower than that in patients with basal ganglia ischemia (ipsilesional S1-M1, p = 0.002; contralesional S1-M1, p = 0.0001, Fig. 7B). In the beta3 band, the Mann–Whitney U test indicated that there was no significant difference in MRPD of bilateral S1-M1 between patients with basal ganglia hemorrhage and basal ganglia ischemia.

**Fig. 8:**
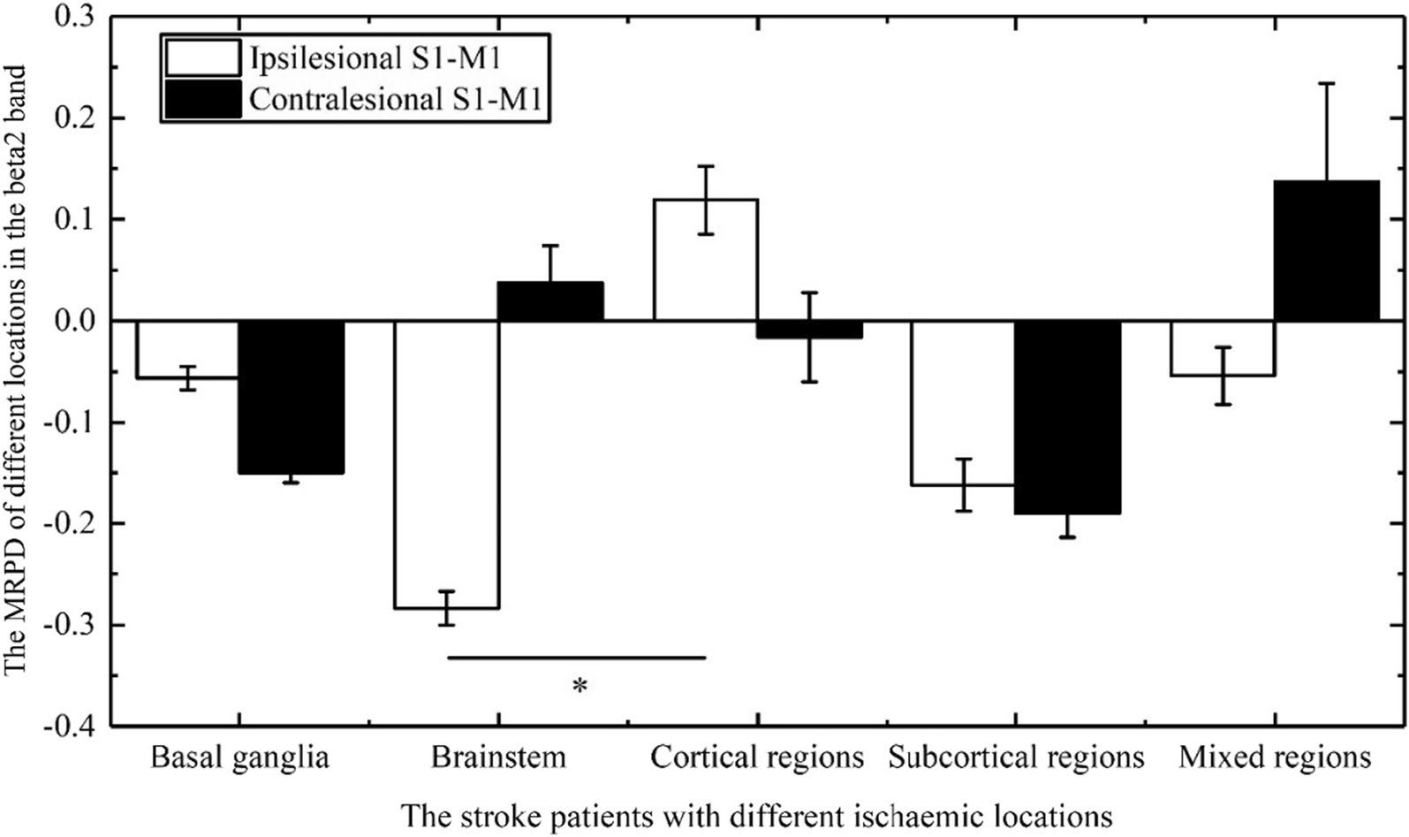
Comparison of MRPD (Motor-related power desynchronization) in the beta2 band induced by FV (Focal vibration) overlying ipsilesional or contralesional S1-M1 in the stroke patients with among basal ganglia ischemia, brainstem ischemia, cortical ischemia, subcortical ischemia, and mixed ischemia. (Asterisks indicate significant differences; *0.01 ≤ *P* < 0.05, **P < 0.01)

In the beta2 band, the Kruskal–Wallis H test revealed that the main factors group for MRPD of ipsilesional S1-M1 had a significance (p = 0.025). Post hoc comparisons revealed that MRPD of ipsilesional S1-M1 in patients with brainstem ischemia was lower than that in patients with cortical ischemia (p = 0.008) (Fig. 8). In the beta1 and beta3 bands, the Kruskal–Wallis H test indicated that the main factors group had no significance for MRPD of both contralesional S1-M1 and ipsilesional S1-M1.

### The association between LC and MBI score

**Fig. 9:**
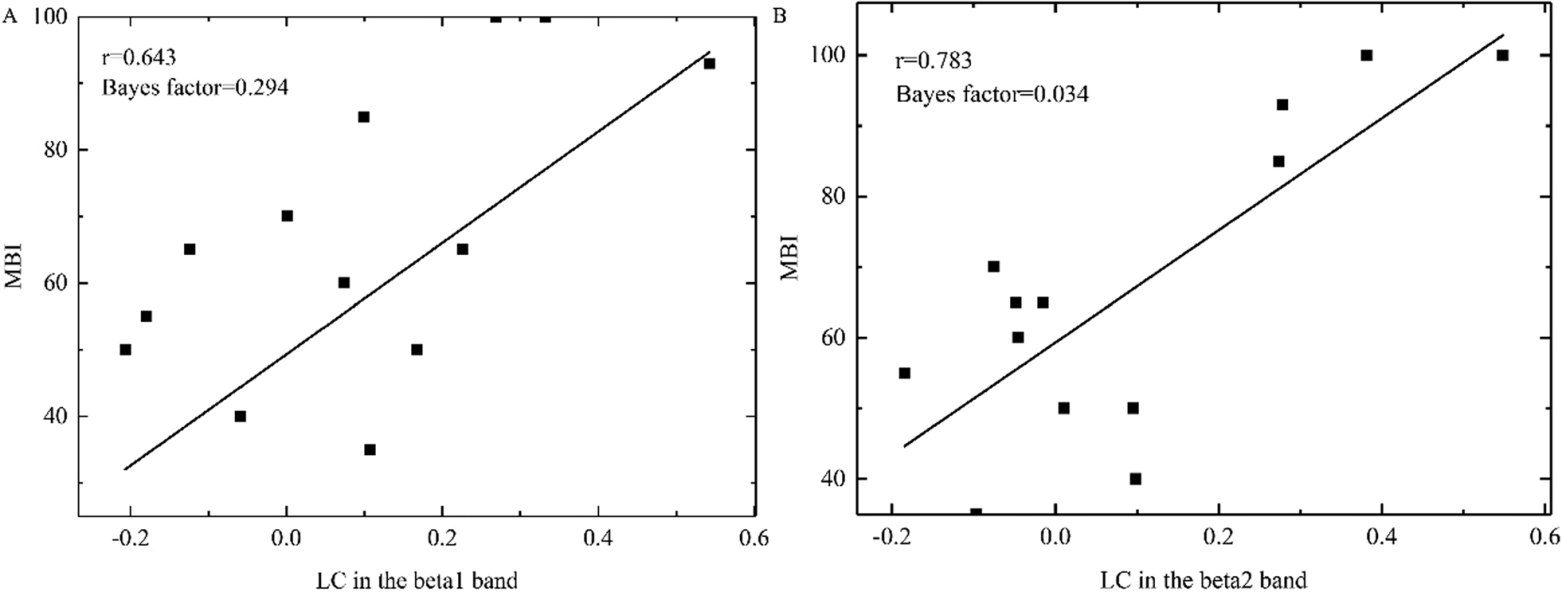
The relationship between MBI (modified Barthel Index) and LC (laterality coefficient) in patients with basal ganglia ischemia in the different bands. **A** shows the correlation between MBI and LC in the beta1 band. **B** shows the correlation between MBI and LC in the beta2 band. Each rectangle represents one stroke subject

The Shapiro–Wilk test showed that LC in all the sub-beta bands, as well as MBI, was normally distributed. When all the stroke patients were  not classified according to the location and the type of damage, Bayesian Pearson correlation analysis showed that there was no evidence for the correlation between LC and MBI in all the sub-beta bands for all the stroke patients. When all the stroke patients were classified according to the location of damage, Bayesian Pearson correlation analysis showed that there was anecdotal evidence for the correlation between LC with MBI in the beta1 band (r = 0.473, Bayes factor = 0.527, Additional file 1: Fig. S2A) and strong evidence for the correlation between LC and MBI in the beta2 band (r = 0.590, Bayes factor = 0.098, Additional file 1: Fig. S2B) in patients with the injuries to the basal ganglia. When all the stroke patients with injuries to the basal ganglia were classified according to the type of damage, in patients with basal ganglia ischemia, Bayesian Pearson correlation analysis showed that there was moderate evidence for the correlation between LC and MBI in the beta1 band (r = 0.643, Bayes factor = 0.294, Fig. 9A), strong evidence for the correlation between LC and MBI in the beta2 band (r = 0.783, Bayes factor = 0.034, Fig. 9B), and anecdotal evidence for the correlation between LC and MBI in the beta3 band (r = 0.562, Bayes factor = 0.654, Additional file 1: Fig. S3). There was no evidence for the correlation between the activation of ipsilesional S1-M1 and MBI, as well as between the activation of contralesional S1-M1 and MBI.

## Discussion

The desynchronized power overlying C3/C4 in the beta band, especially around 20 Hz, can reflect the activation of S1-M1 [32, 36, 37]. Based on this, this study shows the following results: (1) FV may lead to activation of bilateral S1-M1 in patients with basal ganglia hemorrhage/ischemia and other subcortical ischemia, activation of ipsilesional S1-M1 in patients with brainstem ischemia, and no activation of S1-M1 on either side in patients with cortical and mixed ischemia. (2) The activation of ipsilesional/contralesional S1-M1 in patients with subcortical ischemia tends to be higher than that in patients with lesions involving cortical regions. Especially in patients with brainstem ischemia, the activation of ipsilesional S1-M1 could be significantly higher than that in patients with cortical ischemia, while the activation of bilateral S1-M1 in patients with basal ganglia hemorrhage could be higher than that in patients with basal ganglia ischemia. (3) No significant correlation between LC and MBI was observed for all the stroke patients, but a significant correlation between LC and MBI could be observed in patients with basal ganglia lesions.

### The lesion-specific activation patterns of S1-M1 in stroke patients following FV stimulation

1. The lesion-specific activation patterns of S1-M1 among stroke patients with different brain lesion profiles

To the best of our knowledge, this is the first study to explore lesion-specific cortical activation following strong sensory stimulation in patients with subacute stroke. The application of FV at low amplitude (100 μm–2 mm) and high frequency (50–120 Hz) over specific muscles can offer strong proprioceptive inputs by stretching muscle fiber to induce the firing of primary spindle endings [29, 32]. The strong proprioceptive inputs can activate proprioceptive pathways from the muscle spindle, which decussates in the caudal medulla through the posterior column-medial lemniscus pathway, up to S1-M1 [31, 32]. In our study, the frequency of FV (75 Hz) is within this frequency range that can activate the firing of primary spindle endings. The amplitude of FV (1.2 mm) above the firing threshold of muscle spindle primary endings (100 μm) can induce the firing of muscle spindle primary endings [42] and cannot induce the spread of vibration and withdrawal reactions (above 2 mm) [43]. The amplitude of FV is also beyond the amplitude range (0.2–0.5 mm) which can induce kinesthetic illusions [44]. Obviously, FV (frequency: 75 Hz; amplitude: 1.2 mm) is very appropriate to activate muscle spindles. Generally, the limb’s movement can also offer proprioceptive inputs by stretching muscle fibers and activating muscle spindle afferents. Therefore, the activation patterns of S1-M1 induced by FV can be similar to those induced by the movement of limbs. For example, the activation of bilateral S1-M1 in patients with other subcortical ischemia has been confirmed during a single brief handgrip with the affected hand [45], and during hand grasp–release movements [46]. The activation of ipsilesional S1-M1 in patients with brainstem lesions, which has a correlation with the improvement of motor function during motor recovery over 6 months [47], occurred during movement of the affected hand based on fMRI [48]. The activation of ipsilesional S1-M1 can be attributed to the fact that the regeneration and reorganization of the ipsilesional corticospinal tract were detected during recovery in patients with brainstem ischemia [49]. The injuries involving the cortex, including cortical ischemia and mixed ischemia, entail weak activation of ipsilesional or contralesional S1-M1 during a motor task [23, 50], which may be partly explained by the fact that cortical stroke may directly affect S1-M1 regions by inducing extensive and variable locations of lesions [21]. Therefore, compared with traditional motor training alone, additional sensory stimulation, such as FV stimulation, can further promote higher activation of S1-M1, which can induce the plasticity of S1-M1 to a larger extent and finally accelerate motor recovery [51].

In the present study, FV could lead to the different activation patterns of S1-M1 among patients involving injuries to subcortical regions. For example, patients with basal ganglia ischemia show a greater trend than patients with brainstem ischemia in the activation of contralesional S1-M1, which is the opposite in the activation of ipsilesional S1-M1. The difference may be associated with the integrity of the corticospinal tract. Brainstem stroke was associated with significantly lower fractional anisotropy in the contralesional corticospinal tract than capsular stroke [52], which was divided into the basal ganglia [53]. Therefore, the integrity of contralesional corticospinal tract for basal ganglia stroke better than that for brainstem stroke might lead to the difference in the activation of S1-M1 between basal ganglia stroke and brainstem stroke. Moreover, lower fractional anisotropy in the ipsilesional corticospinal tract for capsular stroke than that for pontine stroke might be detected, and hence the activation of contralesional S1-M1 in patients with basal ganglia ischemia may be higher than that in patients with brainstem ischemia [54]. Besides, FV-induced activation of bilateral S1-M1 may be observed in patients with other subcortical ischemia, but not in patients with brainstem ischemia. The abovementioned differences can be attributed to brainstem regions as the common sites in stroke patients involving the motor pathway compared with other subcortical regions [55].

In the present study, the activation of contralesional S1-M1 in patients with basal ganglia ischemia tends to be greater than that in patients with cortical and mixed ischemia, while the activation of ipsilesional S1-M1 in patients with brainstem ischemia is significantly higher than that in patients with cortical ischemia. These results are similar to those of previous studies showing that the activation of S1-M1 in patients with subcortical stroke was higher than that in patients with mixed stroke during paretic movement [22, 56], as well as patients with cortical stroke during different motor tasks [56] or touch discrimination training [21]. The motor recovery can be associated with the activation of S1-M1. Therefore, a poorer motor or somatosensory recovery for cortical stroke than subcortical stroke [9, 57] can support our result. Besides, cortical stroke did not lead to the activation of the motor cortex secondary to critical tissue loss compared with subcortical stroke [20], which can also induce a difference in the activation of S1-M1 between patients with cortical and subcortical stroke.

(2) The lesion-specific activation patterns of S1-M1 between stroke patients with different brain lesion types

FV may induce the activation of bilateral S1-M1 in patients with basal ganglia lesions, which was also observed during the affected hand motion task [53] and during motor recovery 6 months after basal ganglia stroke [58]. However, the effect of brain lesion types on the activation of S1-M1 in the two studies mentioned above has been not explored. Our present study has further demonstrated the activation of bilateral S1-M1 in patients with both basal ganglia hemorrhage and basal ganglia ischemia, where the activation of bilateral S1-M1 in patients with basal ganglia hemorrhage was significantly higher than that in patients with basal ganglia ischemia. Generally, the impairment of motor function in patients with basal ganglia hemorrhage was more serious than that in patients with basal ganglia ischemia [14], but motor improvement in the early stage of rehabilitation in patients with basal ganglia hemorrhage with serious impairment is obviously better than that for patients with basal ganglia ischemia with less impairment [59]. Therefore, patients with basal ganglia hemorrhage may need to raise more neural resources to induce the recovery of motor function compared with patients with basal ganglia ischemia with less severe damage [60]. The activation of bilateral S1-M1 for severely paralyzed stroke patients has been associated with good motor function in the early phase of motor recovery [61], which has also supported our results. Based on our studies, it can be speculated that the activation of S1-M1 in patients with hemorrhage in other brain regions could be higher than that in patients with ischemia in the corresponding brain regions.

### The association between LC and functional scores

In this study, LC shows no significant correlation with MBI for all stroke patients, but LC shows a significantly positive correlation with MBI in patients with basal ganglia lesions. In patients with basal ganglia lesions, the better the performance in activities of daily living gains, the higher the lateralization of contralesional S1-M1. The result is consistent with the previous finding that higher contralesional lateralization is correlated with better motor performance in the subacute phase [53]. One study revealed that sensory stimulation led to the lower activation of ipsilesional S1-M1 in stroke patients with less touch impairment or motor impairment, who can have a good performance in activities of daily living [62]. The lower activation of ipsilesional S1-M1 can lead to higher contralesional lateralization. Therefore, it can be speculated based on this study that patients with good performance in MBI have great contralesional lateralization. Some studies have also demonstrated that the activation of contralesional S1-M1 played an important role in the recovery of motor function [16, 63]. The activation of contralesional S1-M1 could be correlated with the reduction of GABAergic inhibition [64], which can lead to long-term potentiation and cortical plasticity and finally induce the recovery of motor function [65]. Obviously, FV can lead to lesion-specific activation patterns, which can induce lesion-specific mechanisms of cortical reorganization to explain the effect of FV on the recovery of motor function based on the results from those studies [18, 19]. Especially in patients with basal ganglia lesions, optimal activation of bilateral S1-M1 can be achieved by modulation of the FV parameters, including frequency and amplitude. In this way, optimal rehabilitation strategies based on the optimal activation of S1-M1, which is consistent with this EEG-TMS study, can be developed for strong sensory stimulation [66].

### Limitations

The one limitation of this study is that the sample size of stroke patients with different brain lesion profiles is small. Because Fugl–Meyer assessment (FMA) and FMA of upper extremity (FMU) data for some stroke patients were missing, we only investigated the association between LC and MBI. Actually, Bayesian Pearson correlation showed that LC in patients with basal ganglia lesions showed a strong correlation with FMU in the beta2 band when stroke patients without FMU were excluded (r = 0.714, Bayes factor = 0.084, Additional file 1: Fig. S4). Bayesian Pearson correlation showed strong evidence for the correlation between MBI and FMA (r = 0.778, Bayes factor < 0.001, Additional file 1: Fig. S5A) and between MBI and FMU (r = 0.713, Bayes factor < 0.001, Additional file 1: Fig. S5B) when stroke patients without FMU and FMA were excluded. One study has shown that MBI has a positive correlation with FMU or FMA [67], which means that MBI may also be directly used to reflect upper extremity function. Motor functional scores (such as FMU, the Wolf Motor Function Test) and sensory functional scores (such as the revised Nottingham Sensory Assessment scale) can be used to explore the relationship between LC and functional scores in the future. In the current study, we focus mainly on the effect of different brain lesion profiles (including lesion locations and lesion sizes) on the activation of S1-M1, rather than lesion size individually. Further study can focus on investigating the effect of lesion size on the activation of S1-M1 during FV for stroke patients with injuries to the same locations. Besides, broad categorization of stroke types is conducted, which leads to the fact that the sample size of each stroke type is small. Although the current study has demonstrated that the activation patterns of the sensorimotor cortex induced by FV may be related to the brain lesion profiles and lesion types, the relationship between the activation of the sensorimotor cortex and motor functional scores for patients with brainstem ischemia, subcortical ischemia, and other subcortical ischemia, has not been established. The necessity of attempting to discern the relative contributions of stroke lesions to S1 and M1 output may still need to further be explored. Therefore, more patients for each stroke type will be recruited in further studies. Another limitation of this study is that we only compare the activation of the sensorimotor cortex in stroke patients with basal ganglia ischemia and basal ganglia hemorrhage. It is necessary to compare the activation of the sensorimotor cortex in stroke patients with ischemia/hemorrhage in other locations. However, it is very difficult to recruit more hemorrhagic strokes with different lesion locations in the clinical trials. Therefore, in a further study, we should recruit hemorrhagic strokes with different lesion locations (e.g., brainstem) to explore the effects of brain lesion types on the activation of the sensorimotor cortex in subacute stroke patients induced by FV in-depth.

## Conclusions

This study has showed that the activation patterns of S1-M1 induced by FV depended on brain lesion profiles and lesion types. To be specific, the activation of S1-M1 in patients with pure subcortical strokes induced by FV might be higher than that in patients with cortical stroke, while the activation of S1-M1 in patients with basal ganglia hemorrhage induced by FV might be higher than that in patients with basal ganglia ischemia. In addition, better performance in ADL might be found to be associated with a higher lateralization of contralesional S1-M1 in patients with basal ganglia lesions, but not in all stroke patients. This indicates that FV could be applied in a personalized manner based on the lesion-specific activation of S1-M1 in stroke patients with different lesion profiles and types, which may contribute to a better understanding of mechanisms underlying cortical reorganization for heterogeneous stroke patients.

### Supplementary Information

**Additional file 1: Fig. S1.** Comparison of relative power in the beta3 band overlying between ipsilesional S1-M1 and contralesional S1-M1 in the baseline and during-FV (Focal vibration) phase in all the stroke patients. **A** shows the power of ipsilesional S1-M1 significantly lower than that of contralesional S1-M1 in the baseline phase in patients with basal ganglia hemorrhage. **B** shows the power of ipsilesional S1-M1 significantly lower than that of contralesional S1-M1 in the baseline phase in patients with basal ganglia ischemia. **C** shows the power of ipsilesional S1-M1 significantly higher than that of contralesional S1-M1 in the baseline phase in patients with brainstem ischemia. **D** shows the power of ipsilesional S1-M1 significantly lower than that of contralesional S1-M1 in the baseline and during-FV (Focal vibration) phase in with cortical ischemia. **E** shows the power of ipsilesional S1-M1 significantly lower than that of contralesional S1-M1 during FV in patients with mixed cortical–subcortical ischemia. **Fig. S2.** The relationship between MBI (modified Barthel Index) and LC (laterality coefficient) in patients with basal ganglia lesions in the different bands. **A** shows the correlation between MBI and LC in the beta1 band. **B** shows the correlation between MBI and LC in the beta2 band. Each rectangle represents one stroke subject. **Fig. S3.** The relationship between MBI (modified Barthel Index) and LC (laterality coefficient) in patients with basal ganglia ischemia in the beta3 band. **Fig. S4.** The relationship between LC (laterality coefficient) and FMA of upper extremity (FMU) in patients with basal ganglia lesion in the beta2 band. **Fig. S5.** **A** shows the relationship between MBI (modified Barthel Index) and FMA (Fugl–Meyer assessment) when stroke patients without FMA were excluded. **B** shows the relationship between MBI and FMA of the upper extremity (FMU) when stroke patients without FMU were excluded.

## Data Availability

The datasets used or analyzed during the current study are available from the corresponding author on reasonable request.

## Abbreviations

TMS: Transcranial magnetic stimulation
fMRI: Functional magnetic resonance imaging
EEG: Electroencephalography
FV: Focal vibration
LC: Laterality coefficient
S1: Primary sensory cortex
M1: Primary motor cortex
MBI: Modified Barthel index
MRPD: Motor-related power desynchronization
ANOVA: Analysis of variance
FMA: Fugl–Meyer assessment
FMU: Fugl–Meyer assessment of upper extremity

## References

[1] Frenkel-Toledo S, Ofir-Geva S, Soroker N (2020). Lesion topography impact on shoulder abduction and finger extension following left and right hemispheric stroke. Front Hum Neurosci.

[2] Calautti C, Baron JC (2003). Functional neuroimaging studies of motor recovery after stroke in adults: a review. Stroke.

[3] Grefkes C, Fink GR (2020). Recovery from stroke: current concepts and future perspectives. Neurol Res Pract.

[4] Hillis AE, Tippett DC (2014). Stroke recovery: Surprising influences and residual consequences. Adv Med.

[5] Boyd LA, Hayward KS, Ward NS (2017). Biomarkers of stroke recovery: consensus-based core recommendations from the stroke recovery and rehabilitation roundtable. Int J Stroke.

[6] Cheng B, Forkert ND, Zavaglia M (2014). Influence of stroke infarct location on functional outcome measured by the modified rankin scale. Stroke.

[7] Dierick F, Dehas M, Isambert JL (2017). Hemorrhagic versus ischemic stroke: Who can best benefit from blended conventional physiotherapy with robotic-assisted gait therapy?. PLoS ONE.

[8] Frenkel-Toledo S, Fridberg G, Ofir S (2019). Lesion location impact on functional recovery of the hemiparetic upper limb. PLoS ONE.

[9] Ameli M, Grefkes C, Kemper F (2009). Differential effects of high-frequency repetitive transcranial magnetic stimulation over ipsilesional primary motor cortex in cortical and subcortical middle cerebral artery stroke. Ann Neurol.

[10] Lee JH, Kim SB, Lee KW, Kim MA, Lee SJ, Choi SJ (2015). Factors associated with upper extremity motor recovery after repetitive transcranial magnetic stimulation in stroke patients. Ann Rehabil Med.

[11] Lamola G, Fanciullacci C, Sgherri G (2016). Neurophysiological characterization of subacute stroke patients: a longitudinal study. Front Hum Neurosci.

[12] Valdés Hernández MC, Grimsley-Moore T, Sakka E (2021). Lacunar stroke lesion extent and location and white matter hyperintensities evolution 1 year post-lacunar stroke. Front Neurol.

[13] Wei JW, Heeley EL, Wang JG (2010). Comparison of recovery patterns and prognostic indicators for ischemic and hemorrhagic stroke in China: the ChinaQUEST (QUality Evaluation of Stroke Care and Treatment) Registry study. Stroke.

[14] Bhalla A, Wang Y, Rudd A (2013). Differences in outcome and predictors between ischemic and intracerebral hemorrhage: the South London stroke register. Stroke.

[15] Perna R, Temple J (2015). Rehabilitation outcomes: ischemic versus hemorrhagic strokes. Behav Neurol.

[16] Rehme AK, Fink GR, von Cramon DY (2011). The role of the contralesional motor cortex for motor recovery in the early days after stroke assessed with longitudinal FMRI. Cereb Cortex.

[17] Tscherpel C, Dern S, Hensel L (2020). Brain responsivity provides an individual readout for motor recovery after stroke. Brain.

[18] Biasiucci A, Leeb R, Iturrate I (2018). Brain-actuated functional electrical stimulation elicits lasting arm motor recovery after stroke. Nat Commun.

[19] Pundik S, McCabe JP, Hrovat K (2015). Recovery of post stroke proximal arm function, driven by complex neuroplastic bilateral brain activation patterns and predicted by baseline motor dysfunction severity. Front Hum Neurosci.

[20] Luft AR, Waller S, Forrester L (2004). Lesion location alters brain activation in chronically impaired stroke survivors. Neuroimage.

[21] Carey LM, Abbott DF, Lamp G (2016). Same intervention–different reorganization: the impact of lesion location on training-facilitated somatosensory recovery after stroke. Neurorehabil Neural Repair.

[22] Renner CIE, Schubert M, Jahn M (2009). Intracortical excitability after repetitive hand movements is differentially affected in cortical versus subcortical strokes. J Clin Neurophysiol.

[23] Park W, Kwon GH, Kim YH (2016). EEG response varies with lesion location in patients with chronic stroke. J Neuroeng Rehabil.

[24] Rosenkranz K, Rothwell JC (2012). Modulation of proprioceptive integration in the motor cortex shapes human motor learning. J Neurosci.

[25] David JO, Paul LG (2016). Sensory plasticity in human motor learning. Trends Neurosci.

[26] Rosenkranz K, Rothwell JC (2003). Differential effect of muscle vibration on intracortical inhibitory circuits in humans. J Physiol.

[27] Conrad MO, Gadhoke B, Scheidt RA (2015). Effect of tendon vibration on hemiparetic arm stability in unstable workspaces. PLoS ONE.

[28] Paoloni M, Mangone M, Scettri P (2010). Segmental muscle vibration improves walking in chronic stroke patients with foot drop: a randomized controlled trial. Neurorehabil Neural Repair.

[29] Murillo N, Valls-Sole J, Vidal J (2014). Focal vibration in neurorehabilitation. Eur J Phys Rehab Med.

[30] Conrad MO, Scheidt RA, Schmit BD (2011). Effects of wrist tendon vibration on targeted upper-arm movements in poststroke hemiparesis. Neurorehabil Neural Repair.

[31] Marconi B, Filippi GM, Koch G, Giacobbe V, Pecchioli C, Versace V, Camerota F, Saraceni VM, Caltagirone C (2011). Long-term effects on cortical excitability and motor recovery induced by repeated muscle vibration in chronic stroke patients. Neurorehab Neural Re.

[32] Calabro RS, Naro A, Russo M (2017). Is two better than one? Muscle vibration plus robotic rehabilitation to improve upper limb spasticity and function: a pilot randomized controlled trial. PLoS ONE.

[33] Kolbaşı EN, Huseyinsinoglu BE, Bayraktaroğlu Z (2022). Effect of upper limb focal muscle vibration on cortical activity: a systematic review with a focus on primary motor cortex. Eur J Neurosci.

[34] Chen CL, Tang FT, Chen HC (2000). Brain lesion size and location: effects on motor recovery and functional outcome in stroke patients. Arch Phys Med Rehabil.

[35] Andersen KK, Olsen TS, Dehlendorff C (2009). Hemorrhagic and ischemic strokes compared: stroke severity, mortality, and risk factors. Stroke.

[36] Ritter P, Moosmann M, Villringer A (2009). Rolandic alpha and beta EEG rhythms’ strengths are inversely related to fMRI-BOLD signal in primary somatosensory and motor cortex. Hum Brain Mapp.

[37] Lopez S, Bini F, Del Percio C (2017). Electroencephalographic sensorimotor rhythms are modulated in the acute phase following focal vibration in healthy subject. Neuroscience.

[38] Li W, Li C, Ji LH (2019). Study of the activation in sensorimotor cortex and topological properties of functional brain network following focal vibration on healthy subjects and subacute stroke patients: An EEG study. Brain Res.

[39] Li W, Li C, Xu Q, Ji LH (2019). Influence of focal vibration over Achilles tendon on the activation of sensorimotor cortex in healthy subjects and subacute stroke patients. NeuroReport.

[40] Ray AM, Figueiredo TDC, López-Larraz E (2020). Brain oscillatory activity as a biomarker of motor recovery in chronic stroke. Hum Brain Mapp..

[41] Schmider E, Ziegler M, Danay E (2010). Is it really robust?. Methodology.

[42] Fallon JB, Macefield VG (2007). Vibration sensitivity of human muscle spindles and Golgi tendon organs. Muscle Nerve.

[43] Eklund G, Hagbarth KE (1966). Normal variability of tonic vibration reflexes in man. Exp Neurol.

[44] Roll JP, Vedel JP (1982). Kinaesthetic role of muscle afferents in man, studied by tendon vibration and microneurography. Exp Brain Res.

[45] Ward NS, Newton JM, Swayne OBC (2006). Motor system activation after subcortical stroke depends on corticospinal system integrity. Brain.

[46] Kwon YH, Lee MY, Park JW (2007). Differences of cortical activation pattern between cortical and corona radiata infarct. Neurosci Lett.

[47] Ward NS, Brown MM, Thompson AJ (2003). Neural correlates of motor recovery after stroke: a longitudinal fMRI study. Brain.

[48] Kwon YH, Jang SH (2010). Cortical activation pattern in hemiparetic patients with pontine infarct. Eur Neurol.

[49] Zhang M, Lin Q, Lu J (2015). Pontine infarction: diffusion-tensor imaging of motor pathways-a longitudinal study. Radiology.

[50] Ray AM, López-Larraz E, Figueiredo TC (2017). Movement-related brain oscillations vary with lesion location in severely paralyzed chronic stroke patients[C]//2017 39th Annual International Conference of the IEEE Engineering in Medicine and Biology Society (EMBC). IEEE.

[51] Pan LLH, Yang WW, Kao CL (2018). Effects of 8-week sensory electrical stimulation combined with motor training on EEG-EMG coherence and motor function in individuals with stroke. Sci Rep UK.

[52] Guo J, Liu J, Wang C (2019). Differential involvement of rubral branches in chronic capsular and pontine stroke. NeuroImage Clin.

[53] Li Y, Chen Z, Su X (2016). Functional lateralization in cingulate cortex predicts motor recovery after basal ganglia stroke. Neurosci Lett.

[54] Schaechter JD, Perdue KL, Wang R (2008). Structural damage to the corticospinal tract correlates with bilateral sensorimotor cortex reorganization in stroke patients. Neuroimage.

[55] Jiang L, Liu J, Wang C (2017). Structural alterations in chronic capsular versus pontine stroke. Radiology.

[56] Park CH, Kou N, Ward NS (2016). The contribution of lesion location to upper limb deficit after stroke. J Neurol Neurosurg Psychiatry.

[57] Kessner SS, Schlemm E, Cheng B (2019). Somatosensory deficits after ischemic stroke: time course and association with infarct location. Stroke.

[58] Choi JW, Kim MH, Park SA, Sin DS (2017). Neural correlates of motor recovery measured by SPECT at Six months after basal ganglia stroke. Ann Rehabil Med.

[59] Schepers VP, Ketelaar M, Visser-Meily AJ, de Groot V, Twisk JW, Lindeman E (2008). Functional recovery differs between ischaemic and haemorrhagic strokepatients. J Rehabil Med.

[60] Riecker A, Groschel K, Ackermann H, Schnaudigel S, Kassubek J, Kastrup A (2010). The role of the unaffected hemisphere in motor recovery after stroke. Hum Brain Mapp.

[61] van Meer MP, Otte WM, van der Marel K (2012). Extent of bilateral neuronal network reorganization and functional recovery in relation to stroke severity. J Neurosci.

[62] Carey LM, Abbott DF, Harvey MR (2011). Relationship between touch impairment and brain activation after lesions of subcortical and cortical somatosensory regions. Neurorehabil Neural Repair.

[63] Bundy DT, Souders L, Baranyai K (2017). Contralesional brain–computer interface control of a powered exoskeleton for motor recovery in chronic stroke survivors. Stroke.

[64] Muthukumaraswamy SD, Myers JFM, Wilson SJ, Nutt DJ, Lingford-Hughes A, Singh KD, Hamandi K (2013). The effects of elevated endogenous GABA levels on movement-related network oscillations. Neuroimage.

[65] Hensch TK (2005). Critical period plasticity in local cortical circuits. Nat Rev Neurosci.

[66] Schaworonkow N, Triesch J, Ziemann U, Zrenner C (2019). EEG-triggered TMS reveals stronger brain state-dependent modulation of motor evoked potentials at weaker stimulation intensities. Brain Stimul.

[67] Wood-Dauphinee SL, Williams JI, Shapiro SH (1990). Examining outcome measures in a clinical study of stroke. Stroke.

