# Supplementary material for: Lesion-specific cortical activation following sensory stimulation in patients with subacute stroke
Source: J Neuroeng Rehabil. 2023 Nov 13;20:155. doi: 10.1186/s12984-023-01276-8 (PMC10644526; doi:10.1186/s12984-023-01276-8)
Supplement: Supplementary file 1 — Additional file 1: Fig. S1. Comparison of relative power in the beta3 band overlying between ipsilesional S1-M1 and contralesional S1-M1 in the baseline and during-FV (Focal vibration) phase in all the stroke patients. A shows the power of ipsilesional S1-M1 significantly lower than that of contralesional S1-M1 in the baseline phase in patients with basal ganglia hemorrhage. B shows the power of ipsilesional S1-M1 significantly lower than that of contralesional S1-M1 in the baseline phase in patients with basal ganglia ischemia. C shows the power of ipsilesional S1-M1 significantly higher than that of contralesional S1-M1 in the baseline phase in patients with brainstem ischemia. D shows the power of ipsilesional S1-M1 significantly lower than that of contralesional S1-M1 in the baseline and during-FV (Focal vibration) phase in with cortical ischemia. E shows the power of ipsilesional S1-M1 significantly lower than that of contralesional S1-M1 during FV in patients with mixed cortical–subcortical ischemia. Fig. S2. The relationship between MBI (modified Barthel Index) and LC (laterality coefficient) in patients with basal ganglia lesions in the different bands. A shows the correlation between MBI and LC in the beta1 band. B shows the correlation between MBI and LC in the beta2 band. Each rectangle represents one stroke subject. Fig. S3. The relationship between MBI (modified Barthel Index) and LC (laterality coefficient) in patients with basal ganglia ischemia in the beta3 band. Fig. S4. The relationship between LC (laterality coefficient) and FMA of upper extremity (FMU) in patients with basal ganglia lesion in the beta2 band. Fig. S5. A shows the relationship between MBI (modified Barthel Index) and FMA (Fugl–Meyer assessment) when stroke patients without FMA were excluded. B shows the relationship between MBI and FMA of the upper extremity (FMU) when stroke patients without FMU were excluded. [file 12984_2023_1276_MOESM1_ESM.docx]

**Title: Lesion-specific cortical activation followincsensory stimulation in patients with subacute stroke**


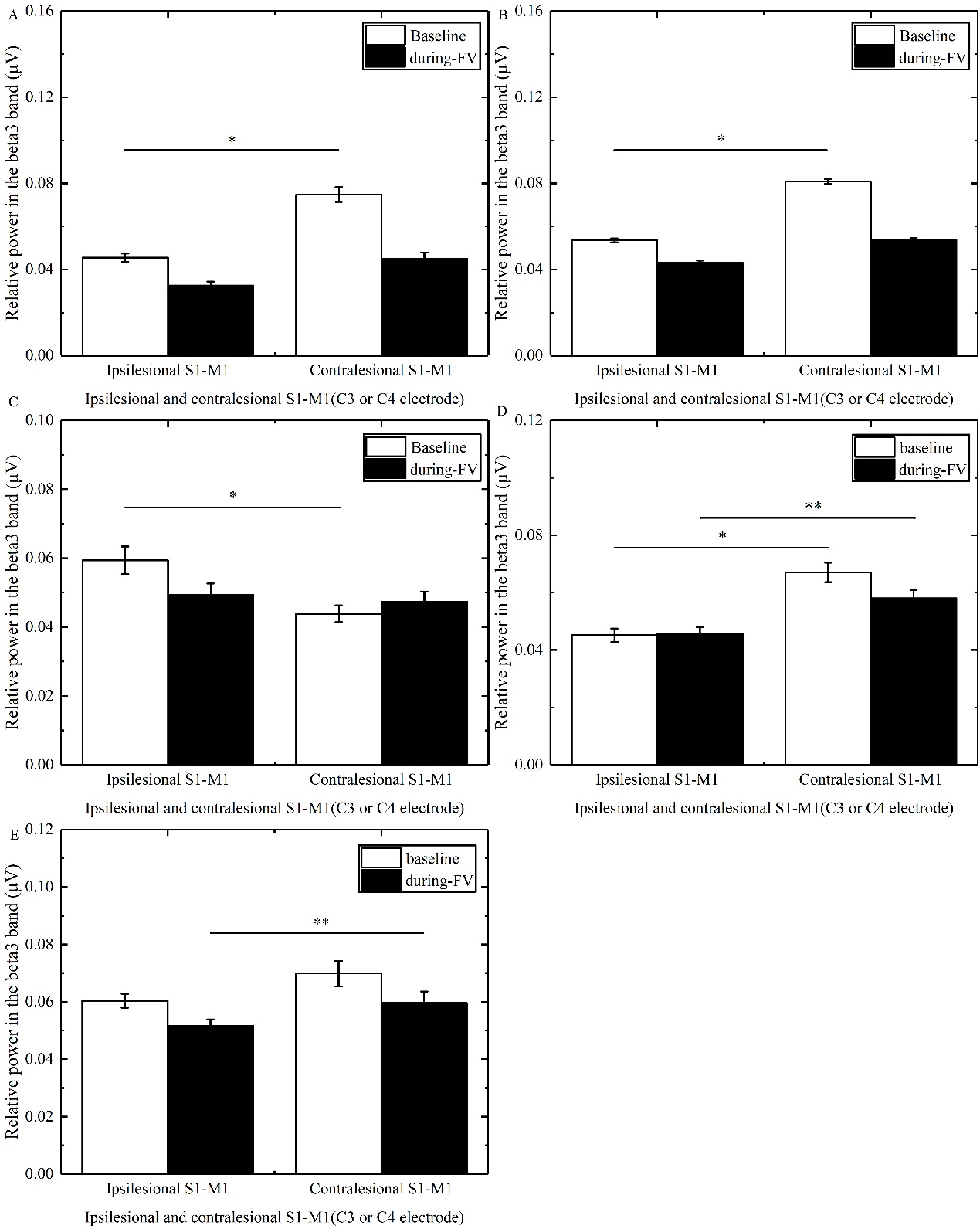


Fig.S1. Comparison of relative power in the beta3 band overlying between ipsilesional S1-M1 and contralesional S1-M1 in the baseline and during-FV (Focal vibration) phase in all the stroke patients. **A** shows the power of ipsilesional S1-M1 significantly lower than that of contralesional S1-M1 in the baseline phase in patients with basal ganglia hemorrhage. **B** shows the power of ipsilesional S1-M1 significantly lower than that of contralesional S1-M1 in the baseline phase in patients with basal ganglia ischemia. **C** shows the power of ipsilesional S1-M1 significantly higher than that of contralesional S1-M1 in the baseline phase in patients with brainstem ischemia. **D** shows the power of ipsilesional S1-M1 significantly lower than that of contralesional S1-M1 in the baseline and during-FV (Focal vibration) phase in with cortical ischemia. **E** shows the power of ipsilesional S1-M1 significantly lower than that of contralesional S1-M1 during FV in patients with mixed cortical–subcortical ischemia (asterisks indicate significant differences; *0.01 ≤ *P* < 0.05, ***P* < 0.01).


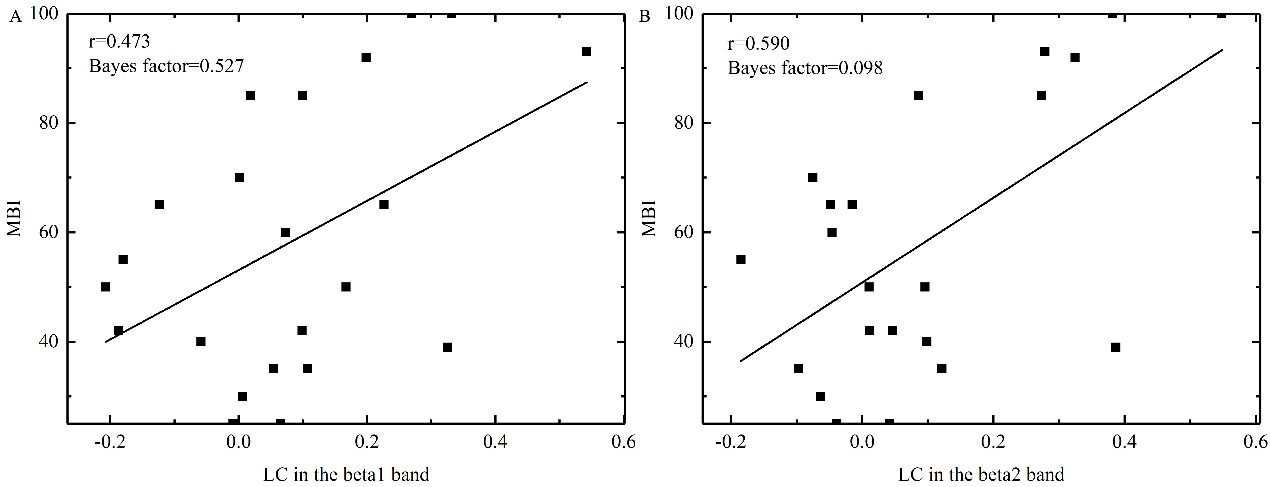


Fig.S2. The relationship between MBI (modified Barthel Index) and LC (laterality coefficient) in patients with basal ganglia lesions in the different bands. **A** shows the correlation between MBI and LC in the beta1 band. **B** shows the correlation between MBI and LC in the beta2 band. Each rectangle represents one stroke subject.


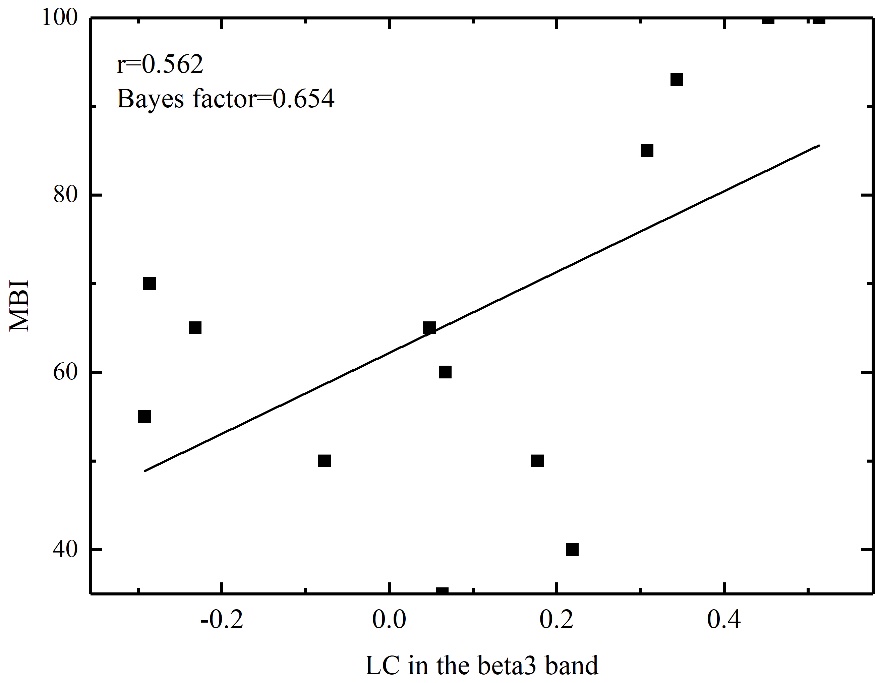


Fig. S3. The relationship between MBI (modified Barthel Index) and LC (laterality coefficient) in patients with basal ganglia ischemia in the beta3 band.


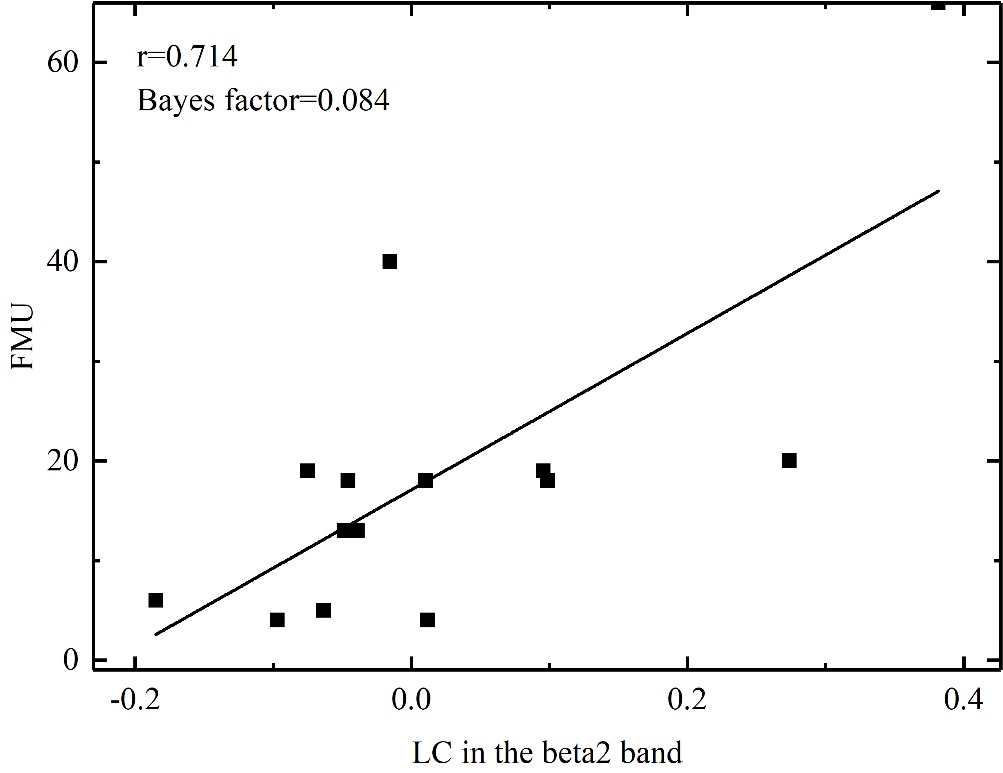


Fig. S4. The relationship between LC (laterality coefficient) and FMA of upper extremity (FMU) in patients with basal ganglia lesion in the beta2 band.


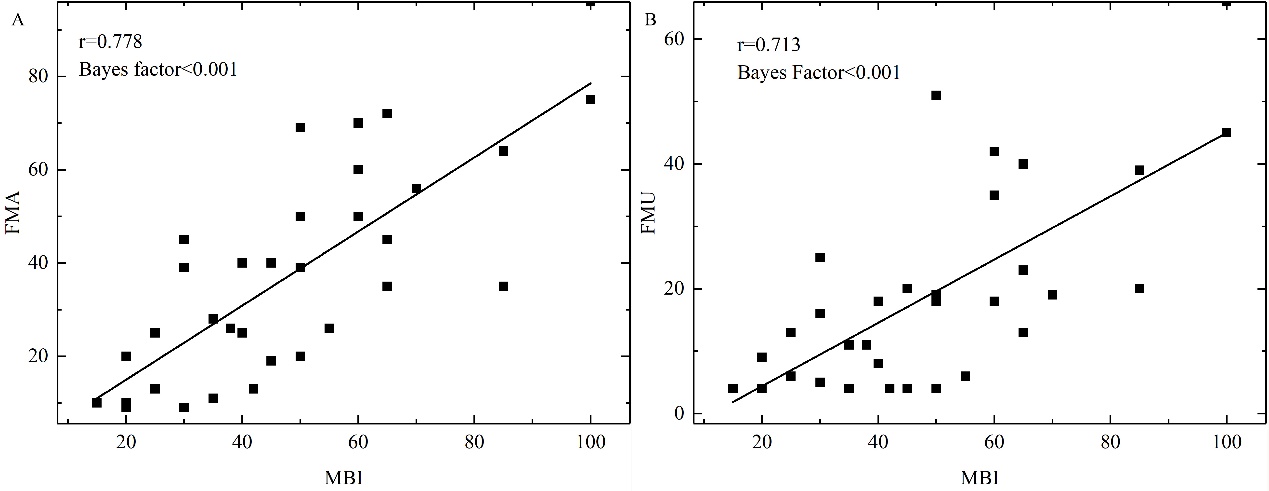


Fig. S5. **A** shows the relationship between MBI (modified Barthel Index) and FMA (Fugl–Meyer assessment) when stroke patients without FMA were excluded. **B** shows the relationship between MBI and FMA of the upper extremity (FMU) when stroke patients without FMU were excluded

.
